# Supplementary material for: Risk Factors for Mortality in Colombian Patients with Candidemia
Source: J Fungi (Basel). 2021 May 31;7(6):442. doi: 10.3390/jof7060442 (PMC8229794; doi:10.3390/jof7060442)
Supplement: Supplementary file 1 [file jof-07-00442-s001.zip › jof-1197406-supplementary.pdf]

Supplementary material to Cortés et al. Risk Factors for Mortality in Colombian Patients With Candidemia:

Table S1. Median age and interquartile ranges of the patients in the different hospitals

Hospital	N	Median age (years)	Interquartile range (years)
Hospital Militar Central	72	33.5	20-64.5
Hospital Universitario San Ignacio	19	43	13-57
Hospital Universitario de la Samaritana	12	58.5	48-70
Fundación Valle de Lili	6	0	0 -0.5

Table S2. Susceptibility results

Species (no. of isolates)	Antifungal	Range	MIC 50	MIC 90	% Non-susceptible
C. parapsilosis (n=42)	Amphotericin	0.25 -1	0.5	1	0
	Anidulafungin	0.25-2	1	2	0
	Fluconazole	0.125-2	0.25	2	2.4
	Voriconazole	0.03-0.25	0.03	0.03	0
C. albicans (n=40)	Amphotericin	0.25-1	1	1	0
	Anidulafungin	0.03-1	0.25	0.25	2.5
	Fluconazole	0.125-4	0.25	0.25	0
	Voriconazole	0.03-0.06	0.03	0.03	0
C. tropicalis (n=19)	Amphotericin	0.25-1	0.5	1	0
	Anidulafungin	0.03-0.5	0.125	0.5	10.5
	Fluconazole	0.125-0.5	0.25	0.5	0

	Voriconazole	0.03-0.06	0.03	0.06	0
C. glabrata (n=5)	Amphotericin	0.5-1	1	1	0
	Anidulafungin	0.03-0.25	0.125	0.25	40
	Fluconazole	2-8	4	8	100
	Voriconazole	0.06-0.5	0.125	0.5	N.D.
Other species (n=3)	Amphotericin	0.25-0.5	0.5	0.5	0
	Anidulafungin	0.06-1	1	1	66*
	Fluconazole	0.125-2	0.125	2	0
	Voriconazole	0.03-0.06	0.03	0.06	N.D

*With *C. guillermondii* break points. N.D.= Not defined
